# Supplementary material for: Are carbon emissions trading and green financial instruments synergistic? -Comprehensive quantitative research based on content analysis
Source: PLoS One. 2024 Mar 7;19(3):e0298601. doi: 10.1371/journal.pone.0298601 (PMC10919601; doi:10.1371/journal.pone.0298601)
Supplement: S1 Data — (ZIP) [file pone.0298601.s001.zip › Data/地级市Z_F工作报告.zip!/╔┬╬≈/╬╝─╧╩╨/└┤╫╘╣1⁄2╓┌║┼í╛┬φ┐╦╩2╛▌═°í┐_2019.docx]

各位代表：

现在，我代表市人民政府向大会报告工作，请予审议，并请市政协委员和列席人员提出意见。

一、2018年工作回顾

2018年，是全面贯彻党的十九大精神的开局之年，也是渭南发展进程中不平凡的一年。面对各种风险挑战和繁重发展任务，全市上下坚持以习近平新时代中国特色社会主义思想为指导，在省委、省政府和市委的坚强领导下，紧扣“追赶超越”定位和“五个扎实”要求，聚焦关中平原城市群次核心城市建设，砥砺奋进、拼搏实干，全力推动高质量发展，经济运行稳中有进，社会大局和谐稳定。生产总值完成1767.71亿元，增长7.1%；全社会固定资产投资增长14.5%；规上工业增加值增长7.2%；地方财政收入实现90.91亿元，增长23.6%；城乡居民人均可支配收入达到31133元和11655元，分别增长8.3%和9.4%。

一年来，我们坚持转型发展，全力聚势能提质量，产业结构更趋优化。倾力推进工业转型、农业提升、旅游升级，加快培育发展新动能。新兴产业快速成长，广汽西北商贸中心开工建设，帝亚纯电动车总装车间主体完工，中国酵素城核心区正式开园，渭南（西安）创新创业孵化器建成投用，战略性新兴产业增加值增长13.9%。传统产业优化升级，龙钢百万吨精品板带、秦悦新型装配式建筑等25个工业项目建成投产，陕煤化新型建材、金钼升级改造等10个技改项目加快推进。非能源工业占比达到72.3%，单位GDP能耗下降4.7%。三产旅游更具活力，成功举办渭华起义90周年纪念活动，深入推进全域旅游示范市创建，新增4A级景区2家，全年接待游客7200万人次，综合收入达到570亿元，均实现35%以上增长。社会消费品零售总额增长10.4%，服务业增加值占比达到41.2%。白水县、蒲城县成功跻身全国电子商务进农村示范县。现代农业稳步提升，粮食总产实现“十四连丰”，全年新增设施农业5.1万亩，新建果园6.79万亩，五大水果区域公用品牌总价值达133亿元。规模养殖场发展到1428个，富平县被评为世界奶山羊产业发展示范区，合阳县被评为全国渔业健康养殖示范县，华阴市被列为全国乡村振兴与特色小（城）镇绿色发展试点。

一年来，我们坚持“项目为王”，全力扩投资增后劲，发展基础更加稳固。深入开展项目攻坚，每季度组织一次集中开工，持续扩大有效投资。350个市级重点项目超额完成投资任务，星美新能源电池、美益特汽车发动机零部件等69个项目建成投产，慈航酵素、新创维产业园等39个项目提前开工，投资规模、增速稳居全省前列。狠抓招商引资“一号工程”，主动出击、精准招商、以质取胜，广汽新能源乘用车整车项目取得重大进展，中关村信息谷、启迪中俄产业园、中航支线飞机等一批新动能项目成功落户，中广核风电、国家光伏应用领跑基地并网发电。特别是单体投资80亿元、年产20万辆的南京金龙纯电动商用车项目，从对接到签约仅用不到3个月时间，创造了招商引资的“渭南速度”。全年共引进项目531个，到位资金突破800亿元。这些项目的落地，必将增强发展后劲，成为助推高质量发展的生力军。

一年来，我们坚持攻坚克难，全力抓整改促提升，三大攻坚战取得积极进展。认真贯彻落实习近平总书记关于秦岭保护重要批示指示精神，制定秦岭生态环境保护规划，完善峪长制等多项管理制度，扎实开展秦岭北麓专项整治，排查发现问题整改完成98%以上，覆土复绿40万平方米。举一反三开展桥山生态治理。铁腕推进治污降霾，突出扬尘整治，实施散煤、“散乱污”企业治理，工业煤炭削减34.66万吨，完成“双替代”7万户。全市环境质量明显改善，成为国务院通报表彰的5个城市之一。澄城县被评为全省铁腕治霾先进县。全力打好精准脱贫攻坚战，紧盯产业扶贫、易地搬迁等重点，集中开展“两对两补”，280个贫困村退出，10.287万人脱贫，富平、蒲城、合阳、澄城4个贫困县脱贫摘帽。全面打响防范化解重大风险攻坚战，以管控政府债务为重点，加强风险预警，规范PPP项目管理，严厉打击非法集资，全市金融风险总体可控。

一年来，我们坚持固本强基，全力提品质塑形象，城乡面貌深刻变化。突出中心城市治理和美丽渭南建设，强健“城市体魄”，扮靓“乡村容颜”。加快补齐基础短板，310国道渭南东至罗敷段改建工程主体完工，合铜高速、黄蒲高速进展顺利，卤阳湖国家民机试飞基地开工建设。围绕“美化、亮化、净化”，全面开展“铺平百姓脚下路、点亮群众头上灯”等10方面综合治理。东风大街、华山大街等提升改造全面完成，金水路北段等6条“断头路”全部打通，渭河综合廊桥等189个城建项目加速推进。实施精细化管理，拆除更换广告牌匾764块，弱电入地11条，亮出了城市美丽天际线。整治人行道障碍物、违规停车场等212处，新改建无害化公厕132座。杜化路、一青里等8条路段夜间出行难题得到解决，城市亮化提升工程全面完工，城市夜景更加绚丽。启动美丽道路、美丽镇街、美丽乡村、美丽经济和美丽文化建设，突出国省干道和通景路绿化提升，新增造林61万亩、绿化道路858公里，建成省级美丽宜居示范村15个，绿树成行、连片成林、环境优美的美丽渭南“种子”茁壮成长。大荔县、合阳县被评为全国“四好农村路”示范县，潼关县被评为全省“四好农村路”示范县，华州区荣获“省级森林城市”称号。

一年来，我们坚持改革创新，全力优环境增活力，集聚效应不断显现。坚持刀刃向内、自我革新，深入推进“放管服”改革，争做金牌“店小二”。积极推行容缺受理、并联审批、多证合一，最大限度减环节、缩流程、降成本，“最多跑一次”办理事项达到525项，企业开办、企业纳税、不动产登记等办理时限大幅压缩。国家工程建设项目审批制度改革试点稳步推进，政府和社会投资类项目审批时限，分别压减至90个和60个工作日以内。全力打造“线上政府”“网上政府”“掌上政府”，渭南政务服务网上线运行。营商环境各项指标大幅提升，新增市场主体4.67万户，增长19.5%。

一年来，我们坚持以人为本，全力保稳定促和谐，民生保障更加有力。全年财政民生支出368.9亿元，占一般公共预算支出的82.7%。实施教育提升五大工程，计划用三年时间、投资121亿元，全力推进城区幼儿园建设、中小学学位补充、改善乡村学校生活设施、加强教师队伍建设和深化教育改革工作。建成投用中小学、幼儿园27所，普惠性幼儿园占比达到76.8%，义务教育段超大班额全部消除。围绕看病难、看病贵问题，启动实施基层千名医生招聘工程，计划用五年时间，为基层招聘1000名医生，首批196人已经上岗。深入推进医药卫生体制改革，分级诊疗县域内就诊率达到94%。城镇新增就业6.4万人，发放各类救助资金5亿元，社会保险持续提标扩面。围绕群众关心关注的热点问题，全力做好供气、供热等民生保障，协调落实气源3.83亿立方米，市内用气紧张矛盾极大缓解。全市第十三届运动会成功举办，体育赛事、文化惠民活动蓬勃开展。国家卫生城市复审顺利通过验收，全国文明城市创建取得阶段性成果。集中开展“大棚房”清理整治和非洲猪瘟防控工作。突出安全生产五项攻坚，深化“平安渭南”建设，扫黑除恶专项斗争综合战绩位居全省第一方阵。

一年来，我们坚持转变作风，全力提效能促实干，自身建设持续加强。严格按照宪法法律履职尽责，自觉接受人大依法监督和政协民主监督，人大代表建议、政协委员提案全部办结。深入开展“讲政治、敢担当、改作风”专题教育，全面推进政府系统党风廉政建设和反腐败斗争，扎实开展“以案促改”，作风效能明显提升。持之以恒落实中央八项规定精神和省、市有关规定，坚决防止“四风”反弹。强化财政预算约束，规范政府采购管理，全市“三公”经费保持只减不增。韩城市被评为全国法治示范市，大荔县、华州区被评为全国法治创建先进县（区）。

同时，国防动员、双拥、国家安全、人民防空、民族宗教、外事侨务等工作不断加强。妇女儿童、老龄、残疾人等事业有了新进步。统计、气象、地震、档案、供销、地方志、新闻出版和广播电视等各项工作取得了新进展。

奋斗充满艰辛，成绩来之不易。这是习近平新时代中国特色社会主义思想科学指引的结果，是省委、省政府和市委坚强领导的结果，是市人大、市政协大力支持的结果，是全市上下锐意进取、团结奋斗的结果。在此，我代表市人民政府，向全市广大干部群众，向各位人大代表、政协委员、离退休老同志，向各民主党派、各人民团体，向驻渭部队官兵，向关心支持渭南发展的各界人士，表示衷心感谢，并致以崇高敬意！

思危方能居安。在追赶超越和高质量发展进程中，全市经济社会发展仍面临一些困难和问题：经济结构不优，调整力度不够，新动能尚需加快培育；投资增长后劲不足，工业投资占比偏低，县域发展不均衡问题突出；民营经济活力不强，营商环境还需优化；打赢三大攻坚战的任务依然艰巨，民生保障与群众期盼还有差距，安全稳定等方面还有不少风险挑战；政府工作还存在标准不高、落实不到位等问题，整治形式主义、官僚主义任重道远。对此，我们必须增强忧患意识、责任意识、担当意识，下非常之功、破非常之难，绝不辜负全市人民期待！

二、2019年工作基本思路和预期目标

今年，是新中国成立70周年，是全面建成小康社会、实现第一个百年奋斗目标的关键之年。政府工作的基本思路是：以习近平新时代中国特色社会主义思想为指导，紧扣“追赶超越”定位和“五个扎实”要求，全面贯彻党的十九大和十九届二中、三中全会精神，坚持稳中求进工作总基调，坚持新发展理念，坚持推动高质量发展，坚持以供给侧结构性改革为主线，坚持深化市场化改革、扩大高水平开放，聚焦建设关中平原城市群次核心城市，继续打好三大攻坚战，叫响“项目为王”，加快产业转型，建设美丽渭南，统筹推进稳增长、促改革、调结构、惠民生、防风险、保稳定工作，以优异成绩庆祝新中国成立70周年。

今年主要预期目标是：生产总值增长7.5%；固定资产投资增长11.5%；地方财政收入增长7%；城乡居民人均可支配收入分别增长8%和9%；城镇登记失业率控制在4.3%以内；万元GDP能耗下降2.6%。

实现全年目标，必须做到“五个紧扣”。要紧扣高质量发展这个基本要求，深入贯彻新发展理念，突出创新驱动引领，加快新旧动能转换，做强做优实体经济。要紧扣以人民为中心这个根本导向，城市建设更贴近民生，社会事业更体现民生，社会治理更重视民生，让群众的幸福感更实在、更充足。要紧扣三大攻坚战这个重大任务，聚焦问题、精准发力、务求实效，拿出硬举措、啃下“硬骨头”，确保脱贫任务全面完成、生态环境有效改善、重大风险总体可控。要紧扣“项目为王”这个有力抓手，聚焦项目建设，狠抓招商引资，以更大的格局谋项目，以更高的眼界引项目，以更拼的干劲争项目，以更强的力度建项目，形成全市上下大抓项目、抓大项目的浓厚氛围。要紧扣县域经济这个重要支撑，强化市域统筹、协同联动，以园区为主战场，推动产业融通、设施联通、要素畅通，提升县域综合实力和竞争力，形成特色彰显、优势互补、齐头迸发的发展新格局。

三、2019年重点工作

今年经济社会发展任务重、挑战多、要求高。我们要坚定信心、保持定力，突出重点、持续发力，扎实做好各项工作。

（一）突出项目支撑，持续增强发展新动能

把项目建设作为加快发展的主抓手，强力度推进，全要素保障，以项目建设实效扩投资稳增长。

集中开展项目攻坚。继续坚持项目工地就是阵地、现场就是考场、进度就是尺度，深入开展“项目攻坚年”活动，突出“六大攻坚”，推动项目建设提速、提质、提效。着力抓好360个市级重点项目建设，力争投资突破千亿元。坚持领导包抓、集中开工、现场观摩，实施项目清单管理，抓协调、保开工、促进度，形成一浪高过一浪的建设热潮。年内渭南热电联产等60个项目竣工投产，启迪中俄产业园、中关村信息谷等项目开工建设，中航支线飞机、中安亚太商用飞机等签约项目尽快落地。加强项目策划储备，完善重大项目库，借“外脑”、引“外智”，提高项目策划水平，高质量谋划包装重大项目300个以上。

强力推进招商引资。始终把招商引资作为“一号工程”，坚持领导带头招商，着力在更新招商理念、创新招商方式、提高招商实效上下功夫。积极适应形势变化，加强产业研究，突破惯性思维，探索“市场+资源+基金+服务”招商新模式，实现招商引资新突破。紧盯国内外500强和行业领军企业，深入开展产业链招商、以商招商和驻点招商，力争一批新能源汽车、智能制造、新材料等产业项目落户渭南。深度参与陕粤港澳经济合作周、丝博会等活动，主动对接、跟踪服务，提高项目落地率和资金到位率。加大招商引资考核力度，定目标、排位次、严奖惩，力争全年招商引资到位资金880亿元。

持续优化营商环境。把优化营商环境作为一场“效率革命”，坚决扫除体制障碍，加快实现“总体省内一流，部分全国领先”目标。持续深化“放管服”改革，再梳理取消下放一批“含金量”高的审批事项，让群众办事像网购一样方便。进一步优化办事流程，让企业多用时间跑市场，少用功夫跑审批。推进“互联网+政务服务”，抓好“线上政府”“网上政府”“掌上政府”建设运营，推行上门服务、“不见面审批”，市县级政务服务事项网上可办率不低于70%。全力做好全国工程建设项目审批制度改革试点，创造更多的渭南经验。优化环保、市场监管检查方式，提升执法监管的法制化、程序化、规范化水平。让良好营商环境涵养项目建设生态、蓄积追赶超越澎湃动能。

（二）突出转型升级，打造产业集聚新引擎

认真贯彻“巩固、增强、提升、畅通”八字方针，做优传统产业，做大新兴产业，以转型发展的强劲态势推动高质量发展。

力推工业转型升级。加快推进新旧动能转换，全年规上工业增加值增长7.5%。改造提升传统产业。聚焦提质增效，推进海泰新材料等32个转型项目建设，完成北人印机等10个技改项目。强化“一企一策”，推动能源化工等传统产业稳产达效。巩固“三去一降一补”成果，加快“僵尸企业”处置，盘活生产要素，拓宽发展空间。大力培育新兴产业。力促帝亚纯电动乘用车产能释放、南京金龙纯电动商用车年内投产，争取广汽乘用车整车制造项目落户。加快天臣新能源项目扩容提质，延长新能源电池产业链，推动动力电池及部件制造深度融入全省汽车产业格局。充分发挥渭南（西安）创新创业孵化器作用，积极实施“西安研发、渭南制造”新模式，争取更多科技成果来渭转化。年内新增规模以上工业企业60户，新兴产业增加值增长13%以上。

加快发展现代农业。全面落实国家粮食安全新战略，切实抓好粮食生产。深化农业供给侧改革，全力推进“果畜菜”产业振兴，增加优质绿色农产品供给，提高“大红袍”花椒、软籽石榴、富平尖柿等产业化水平，新增水果4万亩、设施瓜菜5万亩以上。持续扩大五大水果区域公用品牌效应，再建一二线城市精品直营店6个。加快奶山羊全产业链培育和生猪养殖等5大畜牧业示范区建设，着力打造一批现代农业产业园。加大新型农业经营主体培育力度，提升家庭农场、农民专业合作社规范化水平，年内新增新型经营主体100家。推动农村一二三产业融合发展，壮大农村电商、乡村旅游、民宿经济等新业态，加快实现农业农村现代化。

推进服务业提档升级。坚持以旅游业为龙头，持续释放内需潜力，加快构建以旅游业为主体、多业融合的现代服务业发展格局。着力打造品质旅游，实施旅游品质提升年行动，聚焦“山水城陵红”，出台全域旅游发展规划，加快三级旅游数据中心、集散中心等公共服务体系建设，争创国家全域旅游示范市。擦亮华山金字招牌，对标国际标准，狠抓内涵挖掘和宣传推介，做足山下留客文章。支持华旅集团参与更多景区建设运营，争做区域旅游业发展龙头。加快洽川景区、潼关古城、澄城尧头窑、中华仓颉上古文化园等提质升级，做好中心城区鼓楼、老火车站等历史遗存保护修复，创建一批高A级景区，打造沿黄风情游等一批精品旅游线路。力争全年接待游客7600万人次，旅游综合收入突破600亿元。持续壮大文化产业，突出历史文化和自然元素，促进文化与金融、科技融合发展。全面实施消费升级行动计划，推进国家供应链创新与应用试点，拓展养老、育幼、家政等新型消费，提升陕菜之都品牌效应。办好中甲联赛，做足“体育+”文章，以赛事经济推动服务业大提升。全年社会消费品零售总额增长10%以上。

（三）突出精准务实，赢得三大攻坚新胜绩

把打好“三大攻坚战”放在政府工作首要位置，坚持问题导向，强化精准施策，集中力量攻坚克难，确保取得实效。

坚决打好防范化解重大风险攻坚战。围绕政治和意识形态、经济发展等6个重点领域，强化风险意识，增强防控能力，守住风险底线。落实“坚定、可控、有序、适度”要求，实行政府举债负面清单、终身问责和债务问题倒查机制，坚决遏制增量、积极处置存量，确保全市政府隐性债务有序下降。认真贯彻《政府投资条例》，依法决策、科学决策，确保经济社会发展水平和财政收支状况相适应。坚持堵“后门”与开“前门”并重，既要严堵违规举债，又要用足用活政策，科学合理融资，用发展的手段防范金融风险。加大非法集资打击力度，营造健康有序的金融环境。

坚决打赢精准脱贫攻坚战。把脱贫攻坚作为最大的政治任务，年内实现3.1万人脱贫、27个贫困村退出，白水县完成脱贫摘帽阶段任务。聚焦“两不愁三保障”，突出产业就业扶贫，确保有条件的贫困户至少有1个增收产业，有劳动力的贫困家庭至少1人稳定就业。加快“两房”建设，9月底前易地扶贫搬迁全部达到入住条件。狠抓中省反馈问题整改，高质量完成整改任务。巩固脱贫成果，坚持“四个不摘”，加大政策扶持力度，逐村制定攻坚方案，建立长效脱贫机制。深化苏陕扶贫协作，加大扶智扶志力度，不断激发脱贫内生动力。

坚决打好污染防治攻坚战。牢固树立“绿水青山就是金山银山”理念，深刻汲取秦岭北麓西安境内违建别墅问题教训，重拳整治“五乱”问题，统筹推进秦岭北麓生态治理和桥山生态修复，重塑南北两山天然生态屏障。铁腕实施蓝天保卫战，积极争取国家清洁取暖城市试点，突出扬尘管控，狠抓散煤、“散乱污”企业和“油路车”治理，加快主城区渭化、金钼化学等企业搬迁，确保空气质量持续改善，解决群众“心肺之痛”。全面落实“节水优先、空间均衡、系统治理、两手发力”新时代治水方针，扎实开展脱离实际造景造湖专项治理。全力打赢碧水保卫战，严格落实河湖长制，集中开展河湖“清四乱”专项行动，加强水源地保护和湿地修复，完成7个城镇污水处理厂提标改造，确保重点流域水质稳定达标。强化农业面源污染治理，加快改善土壤环境质量。让秦东大地天更蓝、山更绿、水更清。

（四）突出城乡统筹，加快城镇建设新进程

围绕关中平原城市群次核心城市建设，加快人口集中、产业集聚、功能集成、要素集约，让城市更宜居、县域有活力、乡村更靓丽。

提升中心城市品质。坚持生态、精致、宜居，进一步强基础、塑个性、增活力，不断完善城市功能，兑现城市价值。今年全力抓好渭河综合廊桥等183个重点城建项目建设，突出优质饮水、生态廊道、交通体系三项工作。积极实施优质饮水工程，统筹谋划资源总量和城市容量，科学制定南山支流水资源优化配置方案，努力让更多群众喝上秦岭水。全面推进生态廊道建设，完成渭河、赤水河沿岸和南塬坡面绿化6800亩，加快构筑中心城市绿色屏障。完善城市交通网络，推进209省道渭河特大桥等11个项目建设，着力构建“一轴两环六桥九辐射”交通体系，提升通达能力。实施西南京路、西华街等道路提升改造，全面打通胜利大街西段、六泉路等一批“断头路”，加快补齐供热、供气等基础短板。以全国文明城市创建为抓手，深化城市精细管理，推进弱电入地工程，加快老旧小区改造，持续抓好背街小巷等整治提升，让城市更舒适、更便利。推动华州区加速融入主城区，提高融合度，拓展新空间。

持续壮大县域经济。按照做强产业、做大平台、做优功能的思路，优化城镇布局，突出县城带动，培育特色产业，着力解决县域发展不均衡不协调问题，不断提升县域发展质量和水平。立足资源禀赋、发展潜力，坚持一县一策，强化分类指导，出台加快县域经济发展意见，着力构建主导产业明晰、特色产业突出的现代产业体系。启动县城总体规划修编，加快市域副中心建设，支持县城改善交通、电力等基础设施，继续抓好省市重点示范镇和文化旅游名镇、特色小城镇建设，加快形成以县城为中心、重点镇为骨干的层次清晰、特色鲜明的新型城镇体系。制定支持工业园区发展措施，加大产业引导基金投入力度，加快高新区东区建设，推动澄城经济开发区、白水苹果科技产业园创建省级工业园，努力开创县域园区竞相发展的新局面。

加快美丽渭南建设。以美丽道路为骨架，美丽镇街为节点，美丽乡村为依托，美丽经济为基础，美丽文化为灵魂，全面巩固美丽渭南建设成果，建设生态宜居、彰显特色的新农村。突出道路绿化提升，统筹抓好“四好农村路”建设，年内绿化道路300公里以上，造林45万亩。加快美丽乡村和美丽镇街建设，实施农村人居环境整治三年行动计划，推进垃圾污水处理、厕所革命、村容村貌提升，打造美丽宜居示范村72个，全市60%以上的行政村建成生态村。大力发展美丽经济，推进农业与文化旅游、生态休闲深度融合，让群众吃上“生态饭”“美丽饭”。积极弘扬美丽文化，倡导乡村文明新风，让美丽渭南形象更具魅力。

（五）突出改革创新，有效激发发展新活力

坚定不移深化改革、扩大开放，推动理念提升、服务提升、效率提升，努力把市场活力和社会创造力充分释放出来。

大力发展“三个经济”。坚持强枢纽、扩门户、促流动，建设内外交融、东西共济、全面开放的陕西东大门。年内国家民机试飞基地主跑道基本建成，韩合机场投入运营，华山机场开工建设。力争黄蒲高速蒲白段建成通车、韦罗高速完成年度任务。全力做好西韩城际、合铜高速建设保障，完成西安－潼关、渭南－蒲城－白水轨道交通前期工作，打通经济发展“大动脉”。推进“互联网+物流”建设，支持高新区广汽物流园、经开区商贸物流园争创省级示范物流园区。主动融入“一带一路”建设，持续深化晋陕豫黄河金三角区域协作，积极参与关中协同创新，加快西渭融合、推进富阎一体，促进项目、资金、技术共享对接。加强与国内外友好城市合作交流，不断提升渭南影响力。鼓励各类人才创新创业，为高质量发展提供智力支持和人才保障。

统筹推进重点领域改革。积极稳妥做好新一轮机构改革，构建系统完备、科学规范、运行高效的职能体系。深化收入分配制度改革，建立健全工资增长机制，确保城乡居民收入增长与经济增长基本同步。深入推进国企改革，完善市属企业法人治理结构，积极开展国有资本投资运营试点。推进农村土地制度、集体产权制度改革，鼓励支持土地经营权有序流转。因地制宜开展“三变”改革，发展壮大集体经济。持续抓好庄里试验区等重点改革，推动改革走深走实，更加有效。

全力支持民营经济发展。坚持“两个毫不动摇”，全面落实支持民营经济发展各项政策，深入推进减税降费，创优民营企业发展环境，让民营经济创新源泉充分涌流、创造活力充分迸发。制定支持民营经济发展措施意见，进一步缩减市场准入负面清单，破除“隐性壁垒”，让民营企业轻装上阵、放手发展。积极开展银企对接和企业上市培育，着力解决企业融资难融资贵问题。完善领导干部联系重点企业制度，全力为企业排忧解难，着力构建“亲”“清”新型政商关系。切实维护民营企业家合法权益，在全社会形成尊重、支持、保护民营企业家的浓厚氛围。

（六）突出发展为民，切实增进群众新福祉

坚持以人为本，下大气力办一批群众看得见、摸得着、得实惠的好事实事，不断提升全市人民获得感、幸福感、安全感。

坚持教育优先发展。全面贯彻全国教育大会精神，持续推进教育提升五大工程。今年投资21亿元，新建、改扩建中小学幼儿园49所，新增城区学位5万个以上，引进硕士学历教师200名以上。启动“千园达标”工程，大幅提高普惠性幼儿园占比。着力改善乡村学校生活设施，推进城乡义务教育均衡发展，确保富平县、蒲城县、大荔县通过义务教育均衡发展合格县验收。支持引导规范社会力量兴办教育，加快发展现代职业教育，切实办好人民满意的教育。

加快建设健康渭南。深入推进健康城市、健康县城等8类健康细胞示范建设，全面提升城乡居民健康水平。加快市儿童医院建设，力争市中心医院内科综合楼等建成投用。深入实施基层卫生能力提升三年计划，加大基层千名医生招聘力度，今年再招聘300名医生。持续深化医药卫生体制改革，推进紧密型医联体建设，构建更加成熟稳定的分级诊疗体系，切实缓解群众看病难、看病贵问题。

完善社会保障体系。把稳就业摆在突出位置，落实就业创业政策，全年城镇新增就业5万人以上。推进社会保险提标扩面，完善城乡居民基本医疗保险和大病保险制度，把更多救命、救急好药纳入医保。做好兜底保障、社会救助等工作，关注关爱留守儿童、失独家庭等特殊群体，做到应保尽保。提高退役军人服务管理水平，努力营造“关心国防、尊崇军人”的浓厚氛围。加快公共文化服务设施和县级公共体育场馆建设，实施一批文化体育惠民工程，不断满足群众日益增长的精神文化需求。

维护社会和谐稳定。严格落实安全生产责任制，持续开展道路交通、危险化学品、非煤矿山等安全攻坚行动，坚决遏制重特大生产安全事故。狠抓食品药品监督管理，切实保障人民群众饮食用药安全。继续抓好“大棚房”清理整治和“非洲猪瘟”防控。坚持领导包案下访制度，着力排查化解矛盾纠纷。深入开展“七五”普法，深化“平安渭南”创建，加快“雪亮工程”建设，纵深推进扫黑除恶专项斗争，严厉打击各类“保护伞”“关系网”，持续提升人民群众安全感。

四、全面加强政府自身建设

各位代表！新时代、新要求、新目标，必须有新气象、新担当、新作为。我们要始终坚持党的领导，坚持以人民为中心，不负重托、不辱使命，更好满足人民对美好生活的新期待。

旗帜鲜明讲政治。把政治建设放在首位，把党的领导贯穿政府工作全过程，树牢“四个意识”，坚定“四个自信”，坚决做到“两个维护”。扎实开展“不忘初心、牢记使命”主题教育，提高党员干部政治站位、政治担当和政治自觉。坚决落实中省和市委决策部署，以实际行动践行讲政治根本要求。

依法行政促公平。自觉接受人大监督、政协监督和监委监督，主动接受群众和媒体监督，认真办理人大代表建议和政协提案，让权力在阳光下运行。完善科学民主决策机制，虚心听取社会各界的意见建议，使政府各项决策符合客观实际，更接地气、更合民意。严格按照法定权限和程序行使权力、履行职责，全力打造法治政府。

持之以恒转作风。坚持勤政务实，出实招、办实事、求实效，把更多精力用在抓落实上，确保定下的事干一件成一件。认真落实“基层减负年”要求，持续精文减会，规范督查考核，坚决反对和整治形式主义、官僚主义，切实减轻基层负担。强化责任担当，从严整治不作为、慢作为、乱作为，全力打造服务政府。

从严治政树清风。始终把纪律和规矩挺在前面，严格落实中央八项规定，驰而不息纠治“四风”。加强审计监督，加大公共资金、国有资产、国有资源监管力度，把制度的笼子扎得更紧。坚持全面从严治党，认真落实主体责任，切实履行“一岗双责”，深入推进政府系统党风廉政建设，以零容忍态度严厉惩治腐败。扎实开展“以案促改”警示教育，始终做到警钟长鸣，全力打造廉洁政府。

各位代表，实干托举梦想，奋斗成就未来。让我们更加紧密地团结在以习近平同志为核心的党中央周围，认真贯彻落实习近平新时代中国特色社会主义思想，在市委的坚强领导下，务实担当、积极作为，拼力奔跑、真心追梦，为推动经济社会高质量发展，为实现人民对美好生活的向往，为建设更高品质的美丽渭南而努力奋斗！
